# Effect of VDRA on survival in incident hemodialysis patients: results of the FARO-2 observational study

**DOI:** 10.1186/s12882-015-0006-8

**Published:** 2015-02-06

**Authors:** Piergiorgio Messa, Mario Cozzolino, Diego Brancaccio, Giuseppe Cannella, Fabio Malberti, Anna Maria Costanzo, Umberto di Luzio Paparatti, Vincenzo Festa, Giuliana Gualberti, Sandro Mazzaferro, on behalf of the FARO Study Group

**Affiliations:** Nephrology, Dialysis and renal Transplant, Fondazione Ca’ Granda IRCCS-Policlinico, Milan, Italy; Department of Health Sciences, University of Milan – Renal Division – San Paolo Hospital, Milan, Italy; Dialysis Unit, “Simone Martini”, Milan, Italy; Department of Nephrology, San Martino Hospital, Genoa, Italy; Divisione di Nefrologia e Dialisi, Azienda Ospedaliera Istituti Ospitalieri di Cremona, Cremona, Italy; AbbVie Italy, Campoverde, Latina, Italy; Dipartimento di Scienze Cardiovascolari Respiratorie Nefrologiche e Geriatriche, Sapienza Università di Roma, Roma, Italy

**Keywords:** PTH, Calcitriol, Paricalcitol, Hemodialysis, CKD-MBD

## Abstract

**Background:**

Mortality rate among patients with stage five chronic kidney disease (CKD) maintained on hemodialysis (HD) is high. Although evidence suggests that use of Vitamin D Receptor Activators (VDRA) in CKD patients increases survival, few studies have examined the effect of VDRA in incident HD patients. The FARO-2 study evaluated the clinical outcome of VDRA therapy on mortality in incident HD patients.

**Methods:**

FARO-2 was a longitudinal epidemiological study performed on 568 incident HD patients followed prospectively from 26 dialysis centers over a 3-year period. Data were collected every 6 months using a questionnaire, obtaining clinical, biochemical and therapeutic parameters. Kaplan-Meier curves and Cox proportional hazard regression models were used to determine cumulative probability of time-to-death and adjusted hazard ratios.

**Results:**

568 patients (68% male) with an average age of 65.5 years were followed up. Mean dialysis duration at study entry was 3 months. VDRA use increased from 46% at 6 months to 54.7% at 36 months of follow-up (p = 0.08). No difference was observed in the presence of comorbid diseases at baseline in patients with and without VDRA therapy. Cumulative probability of survival at 24 months was 74.5% (95% CI: 70.2-78.3). Patients receiving VDRA therapy showed a significant increase in survival at 24 months (80.7%; 95% CI: 75.7-84.8) compared to those without (63.3%; 95% CI: 54.8-70.7, p <0.01). The presence of vascular disease, decreased hemoglobin, increased P and lack of VDRA treatment were significantly associated with an increased risk of mortality. Lack of VDRA treatment still remained significant as a predictor of mortality after adjusting for levels of PTH, P and Ca (HR = 2.16, 95% CI: 1.09-4.30, p = 0.03).

**Conclusions:**

Findings from FARO-2 indicate that in incident HD patients VDRA therapy was associated with increased survival.

## Background

Mortality rates among chronic kidney disease (CKD) patients undergoing hemodialysis (HD) is high and exceeds those not undergoing HD [1,2]. The main cause of dialysis related mortality has largely been attributed to cardiovascular disease [2]. CKD patients are also affected by mineral and bone disorders (CKD-MBD), resulting in abnormalities in serum calcium (Ca), phosphorous (P) and parathyroid hormone (PTH). Changes in mineral metabolism have also been associated with higher rates of both all-cause and cardiovascular-related mortality [3-15]. The majority of HD patients are also deficient in the endogenous hormone, 1,25-dihydroxyvitamin D (calcitriol) [16]. The reduction in calcitriol levels, which are a well known causal factor in the pathogenesis of secondary hyperparathyroidism (SHPT) [17], has also been associated with the poor clinical outcomes of these patients [18].

However, the exogenous administration of calcitriol and other vitamin D metabolites may result in hypercalcaemia, potentially contributing to increase the risk of morbidity and mortality [19,20]. To overcome this problem, several synthetic vitamin D receptor activators (VDRAs) have been developed, that are less hypercalcemic and hyperphosphataemic and are efficacious in treating patients with CKD-MBD, [21,22] and as such, are now considered the standard therapy for patients with CKD [23]. In fact, the occurrence of hypercalcemia and hyperphosphatemia is more frequent during calcitriol treatment than with the use of a selective VDRA (paricalcitol), possibly due to a lesser bone resorption and intestinal absorption of these metabolites [24,25]. This may translate into reduced risk of vascular calcifications and hence cardiovascular events. The use of VDRAs, such as calcitriol or the selective VDR activator paricalcitol, is associated with improved survival in HD patients [15,26-32]. It is also recognized that the majority of these retrospective survival studies was performed on prevalent chronic HD patients and it is known that prolonged dialysis is an independent significant predictor of death [33]. To date, only few and small cohort studies have examined the effect of VDRA therapy on survival in incident HD patients. The original FARO study was the first epidemiological study that evaluated SHPT management and survival in HD patients in Italy [32,34]. The aim of the FARO-2 cohort, a sub-group of the original FARO study, was to assess SHPT management and alignment with K-DOQI target ranges, [35,36] and to specifically evaluate the clinical outcome of VDRA therapy on mortality in incident HD patients, using time-dependent Cox models.

## Methods

### Study design

The FARO-2 study was a multicentric epidemiological study performed on a subgroup of patients who initiated the HD therapy over the course of the FARO study (conducted between 2006 and 2007) [32,34]. FARO-2 was undertaken in 26 dialysis centers in Italy. Data collection was retrospective, involving the completion of another two questionnaires for the year of 2008 (added to the previous 4 questionnaires collected during FARO study for the years 2006 and 2007). The physician attending the dialysis procedure completed the clinical and laboratory parameter questions on the dedicated questionnaire for each patient. Final data review and approval were completed by the primary investigator at the study site. Additional information on specific parameters included in the survey has been previously described [34]. All subjects who started dialysis treatment during the FARO study and all subjects at the time of the 1^st^ survey of the FARO Study (April 2006) that had a dialysis vintage of ≤8 months were included. Data are presented by semester from the beginning of dialysis (irrespective of the moment in which the patient entered into the FARO study). The follow up period for each subject is variable and ranged from a minimum of 6 months to a maximum of 3 years. All patients provided written informed consent and the study was approved by all local Ethic Committees.

### VDRA treatment

For patients with stage 5 CKD undergoing HD, the 2003 guidelines of the Kidney Disease Outcomes Quality Initiative (KDOQI) of the National Kidney Foundation were used [35]. According to these guidelines, target ranges for these patients for iPTH concentrations were 150–300 pg/mL and VDRA therapy was only administered to HD patients with iPTH >300 pg/ml. FARO2 was an observational study, therefore medication was administered at the discretion of the treating physician, according to Italian guidelines.

### Statistical analysis

Descriptive statistics of categorical data are presented as summary tables reporting frequencies and percentages, while quantitative variables such as KDOQI parameters were summarized using medians and interquartile ranges. Comparisons between two groups of categorical variables were analyzed by chi-squared tests. Differences in the proportion of patients receiving VDRA therapy over the study period were assessed by chi-squared for trend. Differences for KDOQI parameters were evaluated using Kruskal-Wallis test.

Risk of all-cause mortality was assessed standard survival techniques such as Kaplan-Meier curves to estimate the cumulative probability of death. In particular, an extended Kaplan-Meier survival plot was also performed to assess the effect of VDRA therapy (i.e., untreated vs. oral/intravenous (IV) calcitriol or IV paricalcitol) on all-cause mortality as previously described [15,32]. In brief, those patients not receiving VDRA therapy for the entire follow-up period were always considered as untreated; for those who started VDRA therapy, the risk time for each patient was divided into specific time contributions depending on when treatment started. Before treatment started, the specific time contribution was for the untreated group. As an-intention-to-treat analysis when a patient interrupted or discontinued VDRA they were still considered in the VDRA group. Two multiple time dependent Cox proportional hazard regression models were performed to estimate adjusted HRs (with 95% confidence intervals (95% CI). The first model included various demographic and clinical covariates, including age (per 10-year increase), gender, comorbidities (i.e., hypertension: yes vs. no; dyslipidemia: yes vs. no; heart disease: yes vs. no; neoplasias: yes vs. no; liver disease: yes vs. no; vascular disease: yes vs. no; treatment of diabetes: yes vs. no; haemoglobin (per 1 g/dl increase); albumin (per 1 g/dl increase), VDRA use (untreated vs. treated), calcium-based phosphate binder use (no vs. yes) and non-calcium-based phosphate binder use (no vs. yes). The second model, together with the previously described variables, also included the serum phosphorus (≤3.5 vs. >3.5 to 5.5; >5.5 vs. >3.5 to 5.5 mg/dL), serum calcium (≤8.4 vs. >8.4 to 9.5; >9.5 to 10.5 vs. >8.4 to 9.5; >10.5 vs. >8.4 to 9.5 mg/dL), parathyroid hormone [(PTH) ≤150 vs. 150 to 300; >300 vs 150 to 300 pg/ml]. All parameters were included as time changing variables except age and gender. Patients who moved or were transferred to other dialysis centers or who were lost to follow-up were censored as alive at the last visit performed. To prevent possible non-proportionality of risks among clinical centers, Cox proportional hazard regression models were stratified by clinical center [37]. A *p-*value of <0.05 was considered statistically significant. Statistical analysis was performed using SAS (version 9.2 for WindowsTM, Cary, NC) and STATA (version 12.0, College Station, TX) software.

## Results

### Disposition and baseline clinical characteristics

Patient demographic and clinical characteristics are summarized in Table 1. The proportion of males was higher (68.5%) than females (31.5%). At the start of dialysis, the median age was 70 years for both women and men. The frequency of dialysis sessions was 3 times per week for 83.1% of subjects, twice a week for 12.32% of subjects, once a week for 4.05% of subjects. A small proportion of subjects (0.18%) also had dialysis >3 times per week. Most frequent comorbid diseases included hypertension (77%), ventricular hypertrophy (55.3%) and cardiovascular disease (39.8%). Clinical characteristics remained the same for patients receiving VDRA therapy compared to those without VDRA therapy (Table 1). Furthermore, the presence of comorbid diseases at baseline was similar between the VDRA-treated group (66.9% male; median age 70 years) and non-VDRA treated group (72.1% male; median age 70 years) (Table 1). Although a higher proportion of VDRA-treated patients had hypertension (89.2 with VDRA vs. 83.2 without VDRA, p = 0.05), this difference was not deemed clinically significant. Of the 610 subjects who were considered eligible for participation in the FARO-2 study, 7 subjects were excluded because they were referred to 2 centers that did not adhere to the project while 35 were excluded because, on closer scrutiny, had a dialysis start date earlier than 8 months of the date of study enrollment, therefore not meeting the inclusion criteria. The total sample size was 568 subjects. The number of evaluable patients changed from the 1^st^ survey (128 evaluable patients with a follow-up of 3 years) to the 6^th^ survey (568 evaluable patients with a follow-up of 6 months).

**Table 1:** **Baseline clinical characteristics in patients with and without VDRA therapy**

**Clinical characteristic**	**Total**	**VDRA**		**P-value**
		**Yes**	**No**	
*General*				
N (%)	568	389 (68.5)	179 (31.5)	
Gender (male), n (%)	389 (68.5)	260 (66.9)	129 (72.1)	
Median age (IQR), years	70 (57–77)	69 (56–76.5)	70 (60–77)	0.31
Median height (IQR), cn	165 (160–170)	165 (160–171)	167 (160–170)	0.89
BMI (Kg/M^2^)	23.5 (21.2-26.4)	23.5 (21.2-26.3)	23.4 (20.8-26.5)	0.47
*Comorbid diseases*				
Hypertension, n (%)	496 (87.3)	347 (89.2)	149 (83.2)	**0.05**
Cardiovascular disease, n (%)	338 (59.5)	233 (59.9)	105 (58.7)	0.78
Vascular disease, n (%)	277 (48.8)	188 (48.3)	89 (49.7)	0.6
Diabetes, n (%)	164 (28.9)	109 (28)	55 (30.7)	0.52
Dyslipidemia, n (%)	104 (18.3)	164 (42.2)	61 (34.1)	0.07
Liver disease, n (%)	54 (9.5)	42 (10.8)	12 (6.7)	0.12

BMI = body mass index.

IQR = interquartile range.

Statistically significant p-values are highlighted in bold.

### Levels of PTH, Ca and P at baseline and follow-up

Levels of serum PTH (median values at 6 months vs 36 months; 225 vs 254 pg/ml), Ca (8.8 vs 8.9 g/dl) and P (5.1 vs 4.8 mg/dl) for all patients did not differ significantly from baseline (Table 2). Although median serum PTH levels were significantly different in patients with VDRA compared to those without VDRA therapy (approximately 40% higher in VDRA treated patients), no marked difference was observed for other biochemical (serum calcium and serum phosphorus) and laboratory parameters (serum albumin and hemogobin) (Table 2).

**Table 2:** **Time-dependent characteristics in patients with and without VDRA therapy**

**Characteristic**	**Follow-up time (months)**	**Total**	**VDRA**	**No VDRA**	**p-value**
Serum calcium (mg/dl)	6	8.8	8.8	8.8	0.34
	12	8.9	8.9	8.8	**0.02**
	18	8.9	8.9	8.8	0.26
	24	9.0	9.0	9.0	0.2
	30	9.0	8.9	8.9	0.85
	36	8.9	8.9	9.0	0.11
Serum phosphorus (mg/dl)	6	5.1	5.0	5.2	0.17
	12	5.2	5.2	5.3	0.29
	18	5.0	5.0	5.1	0.44
	24	4.9	4.9	4.9	0.93
	30	4.9	4.9	4.9	0.56
	36	4.8	4.8	4.8	0.49
PTH (pg/ml)	6	225	313	157	**<0.01**
	12	197	245	141.3	**<0.01**
	18	201	227	155	**<0.01**
	24	212	240.3	155	**<0.01**
	30	247	268.3	148.5	**<0.01**
	36	254	260	197.5	0.15
Serum albumin (g/dl)	6	3.60	3.55	3.55	0.09
	12	3.70	3.70	3.60	**0.03**
	18	3.75	3.78	3.70	0.19
	24	3.76	3.80	3.69	0.06
	30	3.74	3.76	3.73	0.51
	36	3.80	3.72	3.81	0.42
Hemoglobin (g/dl)	6	10.9	11.0	10.9	0.08
	12	11.4	11.4	11.5	0.79
	18	11.5	11.4	11.5	0.86
	24	11.3	11.2	11.5	**0.05**
	30	11.2	11.2	11.3	0.23
	36	11.7	11.4	11.9	0.07

PTH = parathyroid hormone. P-values denote comparisons between VDRA and no VDRA groups. Statistically significant p-values are highlighted in bold.

### Use of VDRA

**Figure 1:**
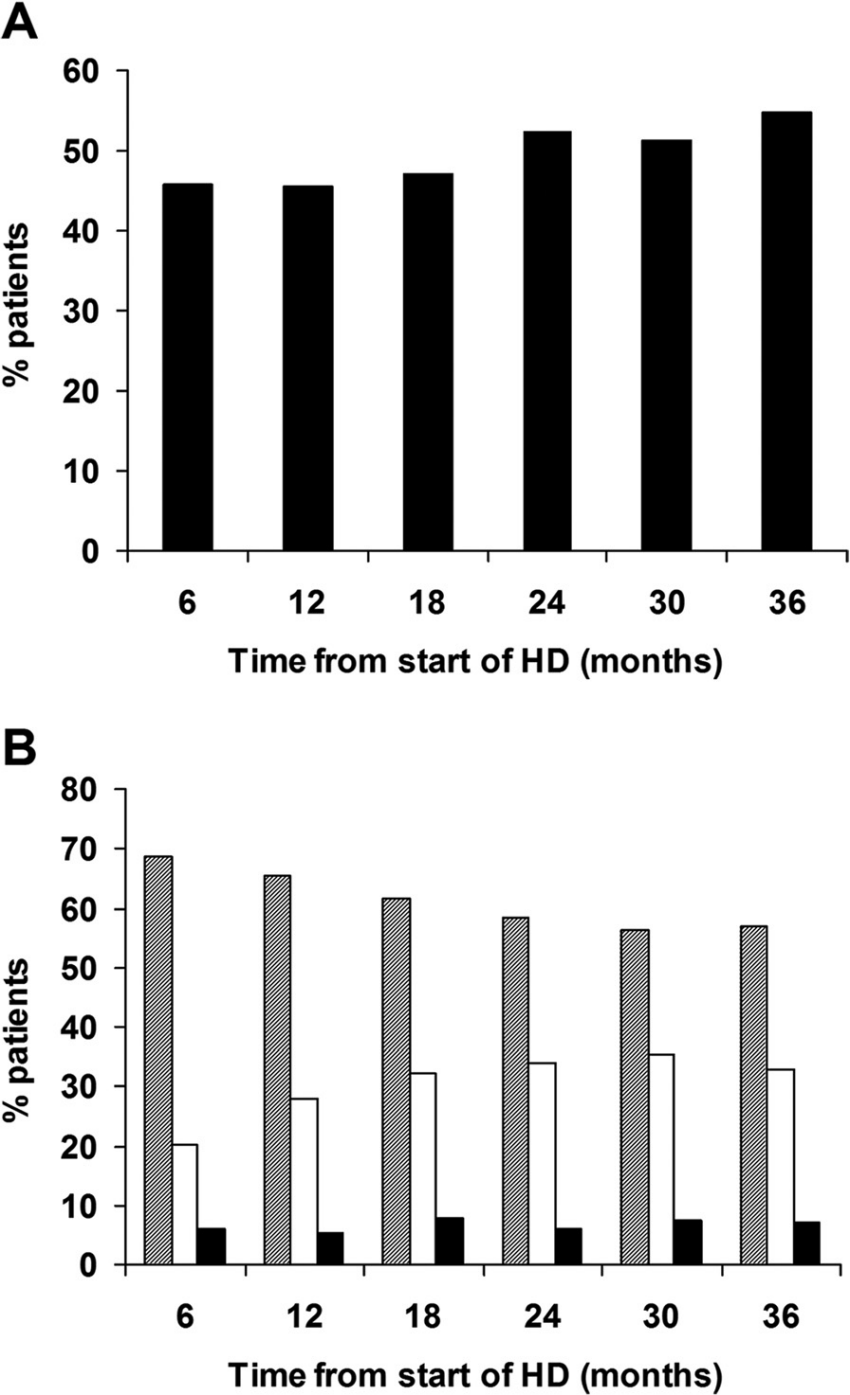
**Proportion of patients receiving VDRA therapy. A)** Proportion of patients receiving any form of VDRA. **B)** Proportion of patients receiving the following various forms of VDRA: oral calcitriol (hatched bars); intravenous paricalcitol (open bars) and intravenous calcitriol (filled bars). Asterix denote statistical significance between 6 and 36 month time points where * = p < 0.05.

389 (68.5%) patients received VDRA therapy at least once during the follow up period and 191 (49.1%) were taken off VDRA(even for a single visit). From the beginning of the study, there was an increase in the use of VDRA from 46% at 6 months to 54.7% at 36 months follow-up, although this increase was not statistically significant (p = 0.08; Figure 1A) (Table 3). Sub-analysis of patients who received VDRA therapy revealed a significant decrease in the use of orally administered calcitriol (68.5% at 6 month follow-up vs. 58% at 36 month follow-up, p = 0.039) and, concomitantly, a significant increase in the use of paricalcitol (IV) (20.9% at 6 months to 33.3% at 36 months, p = 0.045) was observed between baseline and follow up (Figure 1B). Use of calcitriol (IV) was only in a small proportion of patients (<10%) and did not change over the study period (Figure 1B).

**Table 3:** **Concomitant therapy in patients over the follow-up period**

	**Follow-up time (months)**	**Yes**		**No**	
**Treatment**		**N**	**%**	**N**	**%**
VDRA	6	261	46	307	54.0
	12	328	57.8	239	42.2
	18	335	67.5	161	32.5
	24	254	74.1	89	25.9
	30	162	75.7	52	24.3
	36	99	77.3	29	22.7
Non-calcium-based phosphate binders	6	223	39.3	345	60.7
	12	300	52.9	267	47.1
	18	296	59.7	200	40.3
	24	222	64.7	121	35.3
	30	147	68.7	67	31.3
	36	90	70.3	38	29.7
Calcium-based phosphate binders	6	302	53.2	266	46.8
	12	354	62.4	213	37.6
	18	339	68.3	157	31.7
	24	250	72.9	93	27.1
	30	161	75.2	53	24.8
	36	97	75.8	31	24.2

### Concomitant medication

In addition to VDRA use, patients received concomitant medication. At baseline the majority of patients (~70%) received iron supplementation, followed by calcium carbonate (~50%), other phosphate binders (~30%) and insulin (~20%). The proportion of patients receiving medication changed over the study period (Table 3). The proportion of patients receiving non-calcium-based phosphate binders and calcium-based phosphate binders increased from 39.3% at 6 months to 70.3% at 36 months and 53.2% at 6 months to 75.8% at 36 months respectively (Table 3). While the proportion of patients receiving the phosphate binder sevelamer HCl (25.2% vs 38.3%, p = 0.0039), calcium acetate (3.7% vs 10.2%, p = 0.0046) or the calcimimetic cinacalcet (0.9% to 9.4%, p < 0.0001) significantly increased from 6 months to 36 months, the use of iron supplements (73.9% vs 64.8%, p = 0.049) or calcium carbonate (47.4% vs 39.8%, p = 0.15) decreased from 6 months to 36 months.

### Cause of mortality

**Figure 2:**
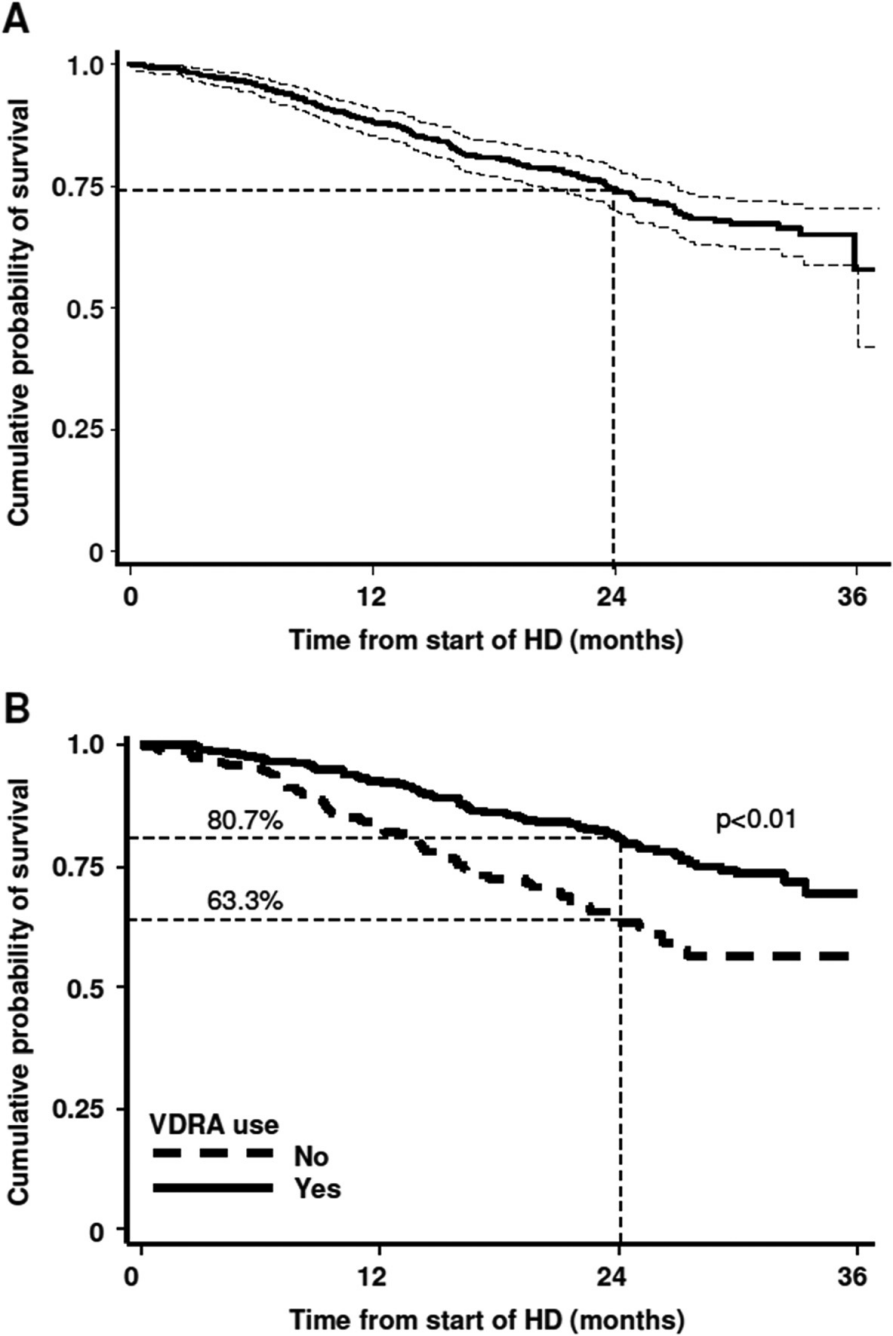
**Kaplan Meier plots showing survival rate of all patients.** The probability of surviving at least 1 year is 88.6% (95% CI: 85.6-91.0), and for at least 2 years is 74.5% (95% CI: 70.2-78.3). Dotted lines represent upper and lower 95% confidence intervals. **A)** Survival estimates (all cause mortality) for FARO-2 patients and **B)** Kaplan Meier plot showing effect of VDRA on survival. Probability of survival at 2 years is indicated. HD = hemodialysis.

In this survey, approximately one quarter (25.2%) of patients died. At 12 months, 47 subjects died (8.3%), at 18 months 44 (16.1%) died, at 24 months 20 (19.6%) died, at 30 months 25 (24%) died and at 36 months 7 (25.2%) died.The main cause of death was heart failure 25.9% (n = 37), followed by other unspecified causes (17.5%), vascular causes and neoplasia (both 16.1%), cachexia (15.4%) and sepsis (9.1%). The cumulative probability of survival (all-cause mortality) from start of dialysis is presented in Figure 2A. Kaplan-Meier analysis revealed that the cumulative probability of surviving at least 12 months was 88.6% (95% CI: 85.6-91.0), at least 24 months was 74.5% (95% CI: 70.2-78.3) (Figure 2A).

### Effect of VDRA on survival

Patients who received VDRA therapy showed a significant higher survival at 24 months (80.7%; 95% CI: 75.7-84.8) compared to those without VDRA treatment (63.3%; 95% CI: 54.8-70.7) (Figure 2B). Actually, the effect of VDRA treatment reveals that the event curves had already separated by 6 months and continued to diverge up to 2 years (Figure 2B).

### Effect of demographic and clinical variables on survival

Cox proportional hazard regression models were used to estimate time to all-cause mortality hazard ratios (HR). The presence of hypertension, vascular disease, decreased hemoglobin and lack of VDRA treatment were significantly associated with an increased risk of mortality (Table 4). Additional analysis adjusted for levels of the biochemical parameters PTH, P and Ca showed that lack of treatment with VDRA still remained significant as a predictor of mortality (HR = 2.16, 95% CI: 1.09-4.30, p = 0.03) (Table 5).

**Table 4:** **Hazard ratios of time to death (any cause), without PTH, Ca and P**

	**HR**	**95% CI**		**p-value**
Age (per 10 year increase)	1.33	0.99	1.80	0.06
Gender (male vs female)	1.22	0.63	2.36	0.55
History of hypertension (yes vs no)	0.32	0.10	0.98	**0.05**
History of dyslipidemia (yes vs no)	0.94	0.42	2.09	0.88
Heart disease (yes vs no)	1.62	0.73	3.58	0.24
Neoplasias (yes vs no)	2.15	0.93	5.00	0.07
Liver disease (yes vs no)	2.04	0.77	5.41	0.15
Vascular disease (yes vs no)	4.60	2.04	10.38	**0.00**
Treatment of diabetes (yes vs no)	1.61	0.71	3.61	0.25
Hemoglobin (per 1 g/dl increase)	0.64	0.49	0.84	**0.00**
Albumin (per 1 g/dl increase)	1.12	0.87	1.45	0.38
VDRA (no vs yes)	2.69	1.38	5.22	**0.00**
Ca-based PiB (no vs yes)	1.11	0.47	2.67	0.81
NoCa-based PiB (no vs yes)	0.58	0.27	1.22	0.15

Data presented as hazard ratios (HR) and upper and lower 95% confidence intervals (CI).

PTH = parathyroid hormone; P = phosphorous, Ca = calcium; VDRA = vitamin D receptor activator; Ca-based PiB = calcium containing phosphate binders; noCa-based PiB = non calcium containing phosphate binders. All variables (but age and gender) were included as time dependent covariates. Statistically significant p-values are highlighted in bold.

**Table 5:** **Hazard ratios of time to death (any cause)**

	**HR**	**95% CI**		**p-value**
Age (per 10 year increase)	1.35	0.98	1.86	0.06
Gender (male vs female)	1.12	0.57	2.21	0.75
History of hypertension (yes vs no)	0.33	0.10	1.07	0.07
History Dyslipidemia (yes vs no)	1.03	0.43	2.45	0.95
Heart disease (yes vs no)	1.38	0.60	3.16	0.45
Neoplasias (yes vs no)	1.89	0.77	4.63	0.16
Liver disease (yes vs no)	1.87	0.64	5.45	0.25
Vascular disease (yes vs no)	4.32	1.90	9.80	**0.00**
Treatment of diabetes (yes vs no)	1.72	0.74	4.01	0.21
Hemoglobin (per 1 g/dl increase)	0.61	0.46	0.81	**0.00**
Albumin (per 1 g/dl increase)	1.08	0.85	1.38	0.54
PTH ≤ 150 vs 150 < PTH ≤ 300 pg/ml	1.02	0.47	2.24	0.96
300 > PTH vs 150 < PTH ≤ 300 pg/ml	0.55	0.19	1.57	0.26
P ≤ 3.5 vs 3.5 < P ≤ 5.5 mg/dl	2.16	0.89	5.26	0.09
P > 5.5 vs 3.5 < P ≤ 5.5 mg/dl	2.63	1.23	5.60	**0.01**
Ca ≤ 8.4 vs 8.4 < Ca ≤ 9.5 mg/dl	1.88	0.90	3.92	0.09
Ca > 9.5 vs 8.4 < Ca ≤ 9.5 mg/dl	1.27	0.44	3.71	0.66
VDRA (no vs yes)	2.16	1.09	4.30	**0.03**
Ca-based PiB (no vs yes)	1.15	0.46	2.89	0.77
NoCa-based PiB (no vs yes)	0.70	0.31	1.58	0.39

Data are presented as hazard ratios (HR) and upper and lower 95% confidence intervals (CI). PTH = parathyroid hormone; P = phosphorous, Ca = calcium; VDRA = vitamin D receptor activator; Ca-based PiB = calcium containing phosphate binders; noCa-based PiB = non calcium containing phosphate binders. All variables (but age and gender) were included as time-dependent covariates. Statistically significant p-values are highlighted in bold.

## Discussion

The main finding of this analysis of the subgroup of the FARO cohort shows that VDRA treatment in incident HD patients (FARO-2 cohort) is associated with reduced overall mortality. This benefit was maintained even after adjusting for levels of the biochemical parameters PTH, P and Ca.

Previous studies have consistently shown benefit in reducing overall and CV-related mortality following VDRA use, compared to non VDRA use, [9,26-32] although studies comparing efficacy of different VDRA molecules (for example paricalcitol compared to calcitriol) on survival outcome have shown differing results [30,38,39].

What makes the FARO-2 study unique is the fact that we have examined the effect of VDRA therapy on survival in incident HD patients, the first study of this kind performed in Italy. Our results confirm that the main cause of death in these patients is attributed to CV-related events [1,2] and that VDRA use may play a beneficial effect on survival in HD patients [9,15,26-32]. In fact, as previously observed in the FARO study, [32] in the present analysis we have also noted that the presence of vascular disease was a significant predictor of mortality. In line with these data, vascular-related comorbidities have previously been linked to increased morbidity and mortality in HD patients [40,41]. There is no clear explanation on the precise mechanisms by which VDRA therapy might provide its cardio-vascular protective effects. To address this, the PRIMO study, a multinational double-blind randomized placebo-controlled trial, examined the effect of paricalcitol (2 μg daily) using cardiac endpoints as measures of CV structure and function (e.g. left ventricular mass) [42]. Although findings from this trial did not demonstrate any effect of paricalcitol on left ventricular mass or improved measures of diastolic function over 48 weeks, this study did show that paricalcitol use was associated with fewer CV-related hospitalizations, with an attenuated rise in BNP levels and decreased left atrial volume [42,43].

Collectively, these findings suggest a possible cardiovascular protective effect of VDRA and in particular of paricalcitol, though the exact mechanisms of this effect still need to be fully elucidated.

### Study limitations

There are, however, some limitations of the FARO-2 study that need to be addressed. Although the number of patients at baseline was relatively large (n = 568), a larger sample size would have permitted additional sub-analysis, accounting for drop-outs and death. The small sample size also precluded the possibility of evaluating the possibility of any potential dose-related effect of VDRA therapy on overall survival. In addition, although some classical factors did not attain statistical significance, possibly again attributed to the low sample number, several other clinical characteristics emerged as risk factors for survival, corroborating other studies [32,40,41]. Furthermore it was retrospective and observational design. On the other hand, there are several strengths of the FARO-2 study worth noting. First, it is based on an observation over a long period of time (36 months) of incident CKD patients undergoing dialysis treatment. Second, it was a multicentric study with the contribution of many centers distributed around Italy and therefore, its findings can be considered well representative of the entire Italian dialysis population. Furthermore, no studies are available reporting on the effect of VDRA therapy on mortality data on incident HD Italian population.

## Conclusions

In summary, the present study suggests that VDRAs may play an important role in decreasing all-cause and cardiovascular mortality in incident HD patients. However, considering the inherent limitations of retrospective analyses, the benefit of VDRA treatment on survival in HD patients still remains to be confirmed in large prospective randomized clinical trials.
